# Benchmark dose modeling for epidemiological dose–response assessment using case‐control studies

**DOI:** 10.1111/risa.17671

**Published:** 2024-11-03

**Authors:** Francesco De Pretis, Yun Zhou, Kan Shao

**Affiliations:** ^1^ Department of Environmental and Occupational Health, School of Public Health Indiana University Bloomington Bloomington Indiana USA; ^2^ Department of Communication and Economics University of Modena and Reggio Emilia, Reggio Emilia Modena Emilia‐Romagna Italy

**Keywords:** Arsenic exposure, Bayesian analysis, Benchmark dose, Bladder cancer, Epidemiological risk assessment, Lung cancer

## Abstract

Following a previous article that focused on integrating epidemiological data from prospective cohort studies into toxicological risk assessment, this paper shifts the focus to case‐control studies. Specifically, it utilizes the odds ratio (OR) as the main epidemiological measure, aligning it with the benchmark dose (BMD) methodology as the standard dose–response modeling approach to determine chemical toxicity values for regulatory risk assessment. A standardized BMD analysis framework has been established for toxicological data, including input data requirements, dose–response models, definitions of benchmark response, and consideration of model uncertainty. This framework has been enhanced by recent methods capable of handling both cohort and case‐control studies using summary data that have been adjusted for confounders.

The present study aims to investigate and compare the “effective count” based BMD modeling approach, merged with an algorithm used for converting odds ratio to relative risk in cohort studies with partial data information (i.e., the Wang algorithm), with the adjusted OR‐based BMD analysis approach. The goal is to develop an adequate BMD modeling framework that can be generalized for analyzing published case‐control study data. As in the previous study, these methods were applied to a database examining the association between bladder and lung cancer and inorganic arsenic exposure. The results indicate that estimated BMDs and BMDLs are relatively consistent across both methods. However, modeling adjusted OR values as continuous data for BMD estimation aligns better with established practices in toxicological BMD analysis, making it a more generalizable approach.

## 1 INTRODUCTION

Benchmark dose (BMD) methodology (Shao & Shapiro, 2018; US Environmental Protection Agency [US EPA], 2012) has become the default approach for determining the toxicity value of chemicals in regulatory risk assessments. Since its introduction (Crump, 1984), the BMD method has evolved into a mature and standardized framework, primarily applied to toxicological data. This framework includes well‐defined input data requirements, various dose–response models, accepted benchmark response (BMR) definitions, and strategies to address model uncertainty (Shao & Shapiro, 2018). These components collectively ensure that BMD analysis of toxicological data is generalizable and interpretable.

However, the application of BMD methodology to epidemiological data presents unique challenges due to the complexities inherent in study designs, exposure measurements, and outcome expressions (e.g., odds ratios [ORs] and relative risks [RRs]). Recent studies have started to bridge this gap by developing strategies to estimate BMDs from epidemiological data. For instance, Kullar et al. (2019) applied BMD methods to individual‐level data for cognitive impairment in children exposed to manganese, while the US Food and Drug Administration (US FDA, 2016) adapted the BMD framework for cohort studies, modeling incidence ratios with adjustments for exposure, and follow‐up durations. Allen et al. (2020b) and Shao et al. (2021) further advanced these methods to handle both cohort and case‐control studies using summary data adjusted for confounders.

Following our previous work that integrated epidemiological data from prospective cohort studies into toxicological risk assessment (De Pretis et al., 2024), this paper shifts focus to case‐control studies. Specifically, it uses the OR as the primary epidemiological measure and aligns it with the commonly used BMD modeling framework for regulatory risk assessment. The objective of this study is to investigate and compare two approaches: the “effective count” based BMD modeling approach (Allen et al., 2020b), combined with the Wang algorithm (Wang, 2013), and the adjusted OR‐based BMD analysis approach (Shao et al., 2021).

This study aims to identify a BMD modeling framework suitable for analyzing published data from case‐control studies. By applying these methods to a dataset examining the association between bladder and lung cancer and inorganic arsenic exposure, we seek to determine the effective approach in terms of consistency and computational efficiency. A significant advancement in this field is the Bayesian BMD methodology, which offers several advantages over traditional methods. Bayesian approaches provide a robust framework for integrating prior information and addressing model uncertainty more effectively. They allow for the incorporation of prior knowledge and the generation of probabilistic statements about model parameters, enhancing the interpretability and reliability of the results. The Bayesian BMD methodology is particularly superior in handling the complexities and uncertainties associated with epidemiological data, making it a powerful tool for regulatory risk assessment.

By focusing on case‐control studies, this research aims to develop a BMD methodology for epidemiological studies that is harmonized with its counterpart for toxicological studies. The selected approach will be evaluated based on its alignment with the established components of a typical BMD modeling framework. Addressing uncertainties in exposure ranges is crucial in epidemiological studies, but outside the scope of this study. High‐quality epidemiological data are preferred over toxicological data for risk assessment because they eliminate the need for animal–to–human extrapolation. Therefore, developing a standardized and generalized BMD modeling framework for epidemiological studies is critical and can significantly impact regulatory risk assessment.

The rest of this article is organized as follows: section 2 describes the arsenic exposure dataset used in our analysis and details the methods for pretreating and analyzing the data using dichotomous and continuous BMD models. In section 3, we compare the models and present the main outcomes of our analysis, exploring their statistical associations. Finally, section 4 discusses the limitations and potential expansions of our approach.

## 2 MATERIALS AND METHODS

The structure and representation of case‐control data used in this study are first introduced in this section to compare two different modeling approaches for BMD estimation from epidemiological studies. The two modeling methods to be compared, model the epidemiological dose–response data (1) as dichotomous data (subsection 2.3.1) or (2) as continuous data (subsection 2.3.2), and are discussed here as well.

### 2.1 Basics for epidemiological data representation

**FIGURE 1:**
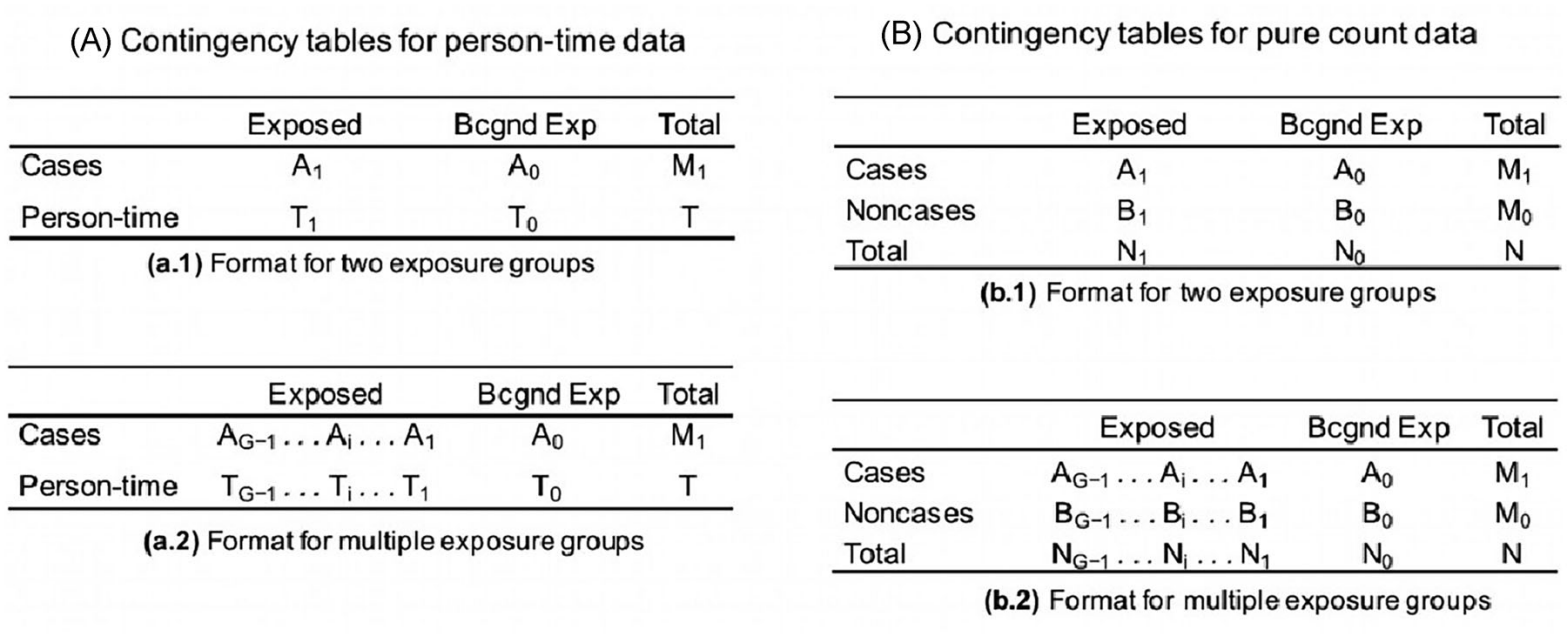
Common notation for contingency tables categorizing epidemiological data. Tables on the left side are employed to represent person–time data, whereas those on the right side are used for pure count data. Examples for two exposure groups or a general number of G groups are provided in sub‐tables.

In line with De Pretis et al. (2024), we make use of the notation introduced in Lash and colleagues (2021, Chapters 16–18) for categorical statistics purposes. We represent absolute frequencies of person–time data and pure count data by the contingency tables (a) and (b) shown in Figure 1. Each of these tables is composed of two sub‐tables, outlining the format used for two exposure groups (exposed and background exposed) and multiple exposure groups (up to a number G). In these two tables, we express cases by the letter *A*, number of controls by the letter *B*, number of subjects by *N*, and person–time by *T*. The latter quantities usually appear as denominators in standard epidemiological ratio‐based measures: we will conform to such notation as well in the formulas provided here. The superscripts *e* and *r* refer to effective and raw (original) counts; they also signal if an epidemiological measure is adjusted or unadjusted. The subscript *i* marks each of the *G* exposure groups, with *i* = 0 denoting the background exposed (Bkgnd Exp, abbreviated) / unexposed / baseline / referent group, as it may be defined in accordance with the context where the latter is used. In this article, we will focus only on case‐control study data type: they are usually characterized by odds ratios, a measure of association between an exposure and an outcome, as it will be now detailed. However, for a more complete description of such studies, we again refer the reader to Lash et al. (2021, Chapter 7).

Similarly to the relative risk RR introduced in Lash et al. (2021, Chapters 16–18), if we consider the number of subjects as the primary denominator in our computations, we favor constructing a Binomial model for the number of cases occurring out of a fixed number of subjects. This leads to defining not only a ratio measure for a given exposure group (the RR), but also a risk‐odds ratio OR, whose maximum likelihood estimate reads as:

(1) $$OR_{i}=\frac{A_{i}}{B_{i}}/\frac{A_{0}}{B_{0}}$$

and with the SE of its logarithmic measure being:

(2) $$\mathrm{SE}\left[\log\left(OR_{i}\right)\right]=\sqrt{\frac{1}{A_{i}}+\frac{1}{B_{i}}+\frac{1}{A_{0}}+\frac{1}{B_{0}}}$$

### 2.2 Dose–response data from case‐control studies

**FIGURE 2:**
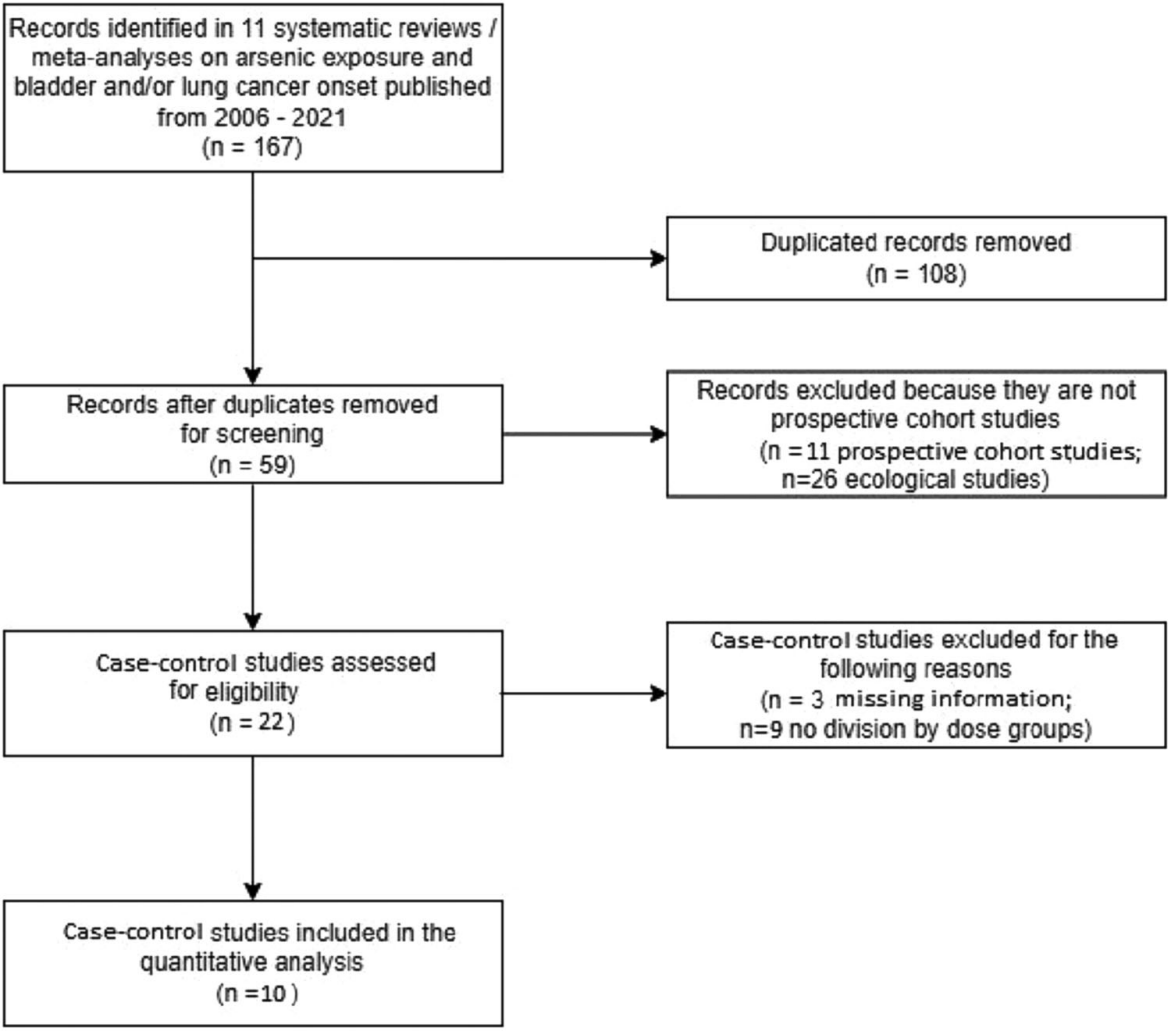
Flowchart for the identification of case‐control studies to be considered for the testing dataset. It maps out the number of records identified, included, and excluded, and the reasons for exclusions.

To compare the results from continuous and dichotomous BMD models (see Subsection 2.3), we consider a list of 11 systematic reviews / meta‐analyses published in 2006–2021 and focused on the coupling between inorganic arsenic exposure by water ingestion and onset of various forms of tumors, principally bladder and lung cancer, since they represent the majority of loci in neoplastic formations recorded in observational studies, next to kidney and liver tumors. Therefore, we limit our analysis to such types of cancer and from these systematic reviews / meta‐analyses we extract data only belonging to case‐control studies (see Figure 2).

Information on extracted data is reported in Table 1: they are all case‐control studies and, differently from De Pretis et al. (2024) which considered data coming only from the Taiwanese area, they account for different geographical exposure zones, such as Bangladesh, Chile, Finland, the United States, and again Taiwan.

**TABLE 1:** Case‐control studies selection.

Systematic Review Meta‐analysis	Case‐ Control Study	Ferreccio et al. (2000)	Ferreccio et al. (2013)	Kurttio et al. (1999)	Mostafa et al. (2008)	Pu et al. (2007)	Smith et al. (2009)	Steinmaus et al. (2013)	Wu et al. (2013)
Allen et al. (2020a)		–	–	–	–	–	–	B, L	B
Boffetta and Borron (2019))		–	–	B	L	–	–	B, L	–
Christoforidou et al. (2013)		–	–	B	–	–	–	–	–
Chu and Crawford‐Brown (2006)		–	–	B	–	–	–	–	–
Lamm et al. (2015)		L	B, L	–	L	–	L	B, L	–
Lamm et al. (2021)		–	B, L	–	–	–	L	–	–
Lynch et al. (2017)		–	–	B	L	B	L	B, L	–
Saint‐Jacques et al. (2014)		L	–	B	–	B	–	B, L	–
Shao et al. (2021)		–	–	B	L	–	L	B, L	–
Tsuji et al. (2014)		–	B, L	B	–	–	–	B, L	–
Tsuji et al. (2019)		–	–	B	L	–	L	B, L	–

*Note*: On the left, the first column shows a list of the principal systematic reviews / meta‐analyses conducted from 2006 to 2021 and related to the binding between arsenic exposure and bladder and lung cancer. The selected case‐control studies are reported in the first row. B (Bladder) and L (Lung) letters mark the studies’ object.

From the same initial list, other 12 studies were reported as case‐control studies but were subsequently discarded, because they did not fit some of the requirements needed for our analysis, resulting in missing information or lacking division by dose groups. Raw data from the selected case‐control studies are shown in the next section, in Tables 2 and 3.

**TABLE 2:** Dose–response odds ratios for case‐control studies and related data for bladder cancer.

Study (Location)	Exposure Concentration Group (ug/L)	Water intake rate (L/day)	Adjusted Exposure Midpoint (ug/L)	Adjusted OR (95% CI)	Cases / Non‐Cases		Number of subjects	
					Raw	Effective	Raw	Effective
Ferreccio et al. (2013) (Chile)	0–59	1.80	26.55	1	23 / 138	19 / 142	161	161
	60–199		116.55	0.84 (0.46–1.52)	27 / 193	22 / 198	220	220
	200–799		449.55	2.50 (1.48–4.22)	60 / 144	51 / 153	204	204
	≥800		1080	4.44 (2.75–7.15)	122 / 165	107 / 180	287	287
Kurttio et al. (1999) (Finland)	<0.1	1.60	0.04	1	26 / 112	13 / 125	138	138
	0.1–0.5		0.24	0.81 (0.41–1.63)	18 / 51	5 / 64	69	69
	>0.5		0.6	1.51 (0.67–3.38)	17 / 51	9 / 59	68	68
Pu et al. (2007) (Taiwan)	≤27.8	2.23^*^	31.0	1	24 / 104	18 / 110	128	128
	27.8–61.0		49.51	1.90 (1.10–3.40)	44 / 104	35 / 113	148	148
	>61.0		102.02	5.30 (3.10–9.00)	109 / 105	99 / 115	214	214
Steinmaus et al. (2013) (Chile)	<26	1.80	11.7	1	33 / 202	14 / 221	235	235
	26–79		47.25	0.92 (0.52–1.61)	33 / 189	12 / 210	222	222
	80–197		124.65	2.62 (1.53–4.50)	71 / 142	30 / 183	213	213
	>197		265.95	6.00 (3.38–10.64)	95 / 107	56 / 146	202	202
Wu et al. (2013) (Taiwan)	<15.5	2.23^*^	8.64	1	44 / 196	27 / 213	240	240
	15.5–42.5		32.34	1.42 (0.90–2.25)	63 / 196	40 / 219	259	259
	>42.5		71.08	4.13 (2.69–6.35)	192 / 202	135 / 259	394	394

* Based on EPA latest report supplementary material for Taiwan (US EPA, 2023).

**TABLE 3:** Dose–response odds ratios for case‐control studies and related data for lung cancer.

Study (Location)	Exposure Concentration Group (ug/L)	Water intake rate (L/day)	Adjusted Exposure Midpoint (ug/L)	Adjusted OR (95% CI)	Cases / Non‐Cases		Number of subjects	
					Raw	Effective	Raw	Effective
Ferreccio et al. (2000) (Chile)	0–10	1.80	4.5	1	6 / 70	5 / 71	76	76
	10–29		17.55	1.70 (0.50–5.10)	9 / 68	8 / 69	77	77
	30–49		35.55	3.90 (1.20–13.40)	7 / 24	7 / 24	31	31
	50–199		112.05	5.50 (2.20–13.50)	52 / 130	51 / 131	182	182
	200–400		270	9.00 (3.60–22.00)	77 / 127	79 / 125	204	204
Ferreccio et al. (2013) (Chile)	0–59	1.80	26.55	1	48 / 138	32 / 154	186	186
	60–199		116.55	0.77 (0.49–1.21)	52 / 193	34 / 211	245	245
	200–799		449.55	1.38 (0.89–2.13)	69 / 144	48 / 165	213	213
	≥800		1080	2.39 (1.61–3.54)	137 / 165	100 / 202	302	302
Mostafa et al. (2008) (Bangladesh)	0–10	3.50	8.75	1	354 / 186	109 / 431	540	540
	11–50		52.5	1.13 (0.91–1.40)	1303 / 576	128 / 448	1879	576
	51–100		131.25	1.28 (0.92–1.77)	208 / 84	71 / 221	292	292
Smith et al. (2009) (USA)	0–9	1.80	4.05	1	11 / 92	8 / 95	103	103
	10–59		31.05	0.70 (0.30–1.70)	7 / 81	5 / 83	88	88
	60–199		116.55	3.40 (1.80–6.50)	35 / 87	27 / 95	122	122
	200–399		269.55	4.70 (2.00–11.00)	23 / 44	19 / 48	67	67
	400–699		494.55	5.70 (1.90–16.90)	11 / 12	7 / 16	23	23
	700–999		764.55	7.10 (3.40–14.80)	64 / 103	62 / 105	167	167
Steinmaus et al. (2013) (Chile)	<26	1.80	11.7	1	61 / 202	21 / 242	263	263
	26–79		47.25	0.98 (0.62–1.53)	61 / 189	20 / 230	250	250
	80–197		124.65	1.70 (1.05–2.75)	85 / 142	29 / 198	227	227
	>197		265.95	3.18 (1.90–5.30)	99 / 107	45 / 161	206	206

### 2.3 BMD modeling methods for epidemiological data

There are four parts in this subsection: we provide a description of the models we employ to analyze dichotomous and continuous data in the first two parts respectively, and then focus on the pre‐treatment of the dose (adjusted exposure midpoint computations), and of the response (BMR calibration).

#### 2.3.1 Model epidemiological data as dichotomous data

This model works in two steps. Initially, data are pre‐treated to derive the “*effective counts*” based on an approach following Allen et al. (2020b) and the Wang algorithm (Wang, 2013), that is, the effective number of cases *A* and of controls *B* obtained as we consider the OR and the interval of its SEs both varying with respect to the different dose groups. Eventually, the effective counts are modeled as dichotomous data to calculate BMDs as outlined in Shao and Shapiro (2018).

**Effective Counts via Wang algorithm**: We first set the number of subjects to be invariant between raw and effective counts, in line with Allen et al. (2020b):

(3) $$N_{i}^{e}=N_{i}^{r}$$

Then, we compute the number of effective counts for the referent group via the Wang algorithm, in the following manner:

(4) $$\left(A_{0}^{e};B_{0}^{e}\right)=\min_{\left(A_{0}^{e};\;\mathrm{SS}\right)}W\left(N_{0}^{r},N^{r},{\bar{\mathrm{OR}}}^{r},{\bar{\mathrm{OR}}}_{L}^{r},{\bar{\mathrm{OR}}}_{U}^{r}\right)$$

where *W* symbolizes the Wang algorithm, *N* is the total number of subjects counting for all dosage groups, the bar symbol operator represents the mean over all the odds ratios and their lower and upper bound of the confidence interval at the 95% level, and SS is a sum of squares as defined in Wang (2013). Eventually, we derive the full number of effective counts for the treatment groups (that is, for all remaining dosage groups) by exploiting the previously mentioned condition on the number of subjects and, similarly, an equivalence of ORs between raw and effective counts is also derived:

(5) $$\left\{\begin{array}{c} N_{i}^{r}=A_{i}^{e}+B_{i}^{e}\\ {\mathrm{OR}}_{i}^{r}=\frac{A_{i}^{e}}{B_{i}^{e}}/\frac{A_{0}^{e}}{B_{0}^{e}} \end{array}\right.$$

**Bayesian benchmark dose modeling for dichotomous data**: In this case, we couple the Allen et al. (2020b) and Wang (2013) “effective counts” method with the dichotomous case of the Shao and Shapiro (2018) model. The latter computes the dose–response model parameters by estimating the following quantity:

(6) logPdata|θ=∑i=0G−1logniyi+yilogfdi|θ+ni−yiniyilog1−fdi|θ

where, ***θ*** represents the parameters that define a dose–response curve *f*(*d_i_* │ ***θ***) (for our comparison purposes, we will focus on the quantal‐linear and dichotomous Hill models for dichotomous data), *d_i_* represents the dose level; *n_i_* is the number of subjects in each dose group (i.e., $N_{i}^{e}$) and *y_i_* is the number of subjects showing response in the corresponding dose group (i.e., $A_{i}^{e}$). Unlike its original version, the summation index is defined from *i* = 0 to *i* = *G*‐1 since the referent group is marked here by having *i* = 0.

With respect to input data, to incorporate the “effective counts” treatment in a dichotomous model, it appears natural to set $\;n_{i}=N_{i}^{e}$ and $y_{i}=A_{i}^{e}$. However, it must be noted that the *n_i_* and *y_i_* terms may no longer be integers, after undergoing such a transformation. To counteract such a problem, as in De Pretis et al. (2024), we consider a classical extension to two real valued arguments through the Gamma function (for instance, see Díaz and Cano (2019); Winkelmann (2008)). An approximation to the closest integer can performed as well.

#### 2.3.2 Model epidemiological data as continuous data

The second way we model epidemiological dose–response data and compare with the approach described in Section 2.3.1 is to model the OR as a continuous response following a typical BMD modeling framework. There are four required input quantities for performing a BMD modeling using continuous data, including dose or exposure levels, the number of subjects in each dose group, the mean, and standard deviation of the response in each dose group. The method described in Section 2.3.3 can be used to derive a reasonable point estimate for each exposure group if the exposure was reported in ranges not as point estimates in the original studies. The sample size of subjects in each exposure group is typically reported in epidemiological studies and can be directly used in BMD modeling. Then, we need to convert the OR (typically reported as median and 95% confidence interval) to mean and standard deviation to facilitate the BMD modeling as continuous data. Usually, reported metrics of OR show that the confidence interval is skewed to the upper end, indicating that it is reasonable to assume that OR at each exposure level follows a lognormal distribution. The lognormal distribution can be characterized by two parameters, $\bar{y_{i}}=\log\left(OR_{i}^{e}\right)$ (i.e., the logarithm of the median OR) and $s_{i}^{\prime }$ (i.e., the standard deviation of OR on a log scale). Based on the reported confidence interval of OR, the $s_{i}^{\prime }$ can be calculated as ${\left(2\cdot z_{0.975}\right)}^{-1}\cdot \log\left(OR_{i}^{U}/OR_{i}^{L}\right)$ for 95^th^ percentile CI (note that z_0.975_ needs to be replaced by z_0.95_ if 90^th^ percentile CI was reported in the original study). Then, as described in Shao et al. (2021), these four required quantities will be used in the following log‐likelihood function to estimate the parameters of a continuous dose‐response model:

(7)

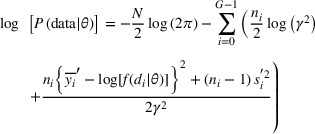

where *n_i_* is the number of subjects in each dose group, *d_i_* is the exposure level of each group, ${\bar{y_{i}}}^{\prime }$ is the log‐transformed mean value of OR in each group, $s_{i}^{\prime }$ is the log‐transformed standard deviation of OR in each group, *N* is the total number of subjects, and *G*−1 is the number of dose groups. *f*(*d_i_* | ***θ***) represents a continuous dose–response model with a vector of parameters ***θ***. Based on the settings expressed in the log‐likelihood function above, we assume that the mean response of OR on the log‐scale is represented by a chosen continuous dose–response model, and the within‐dose–group standard deviation, *γ*, is a constant across the dose groups. In this study, for the purpose of comparison, one simple and one complex dose–response model (i.e., the Linear model and the Hill model as described in Shao and Shapiro [2018] corresponding to the quantal‐linear and dichotomous Hill models for dichotomous data) are used for BMD estimation.

#### 2.3.3 Point exposure computations

Point estimate of exposure/dose level (instead of exposure/dose ranges) is required to be used in a typical BMD modeling framework. To ensure the comparisons among different epidemiological studies tackling diverse populations are effective and consistent, adjustment to the exposure metrics has to be taken into account as well. For example, Lynch et al. (2017) estimated the midpoint arsenic concentration in water of the dose groups, adjusted to account for differences in body weight and water consumption rates in some foreign populations as compared to the United States. To estimate the midpoint exposures for open‐ended highest dose groups presented as greater than a value, Lynch et al. (2017) assumed the midpoint between the highest value and two times of the highest value. If an open‐ended lowest dose group exists (i.e., less than a lowest dose), then it is simple to use half of the lowest value as the midpoint for the lowest group. This approach can be formalized in the following way. For the datatype of dose ranges, $\delta_{i}^{*}$ and $\delta_{i}^{◇}$ represent the supremum and infimum (shortly, *sup* and *inf*) of a given dose interval referring to the *i*‐th group, respectively. Adjustment is made based on the assumption that a water intake rate *ω* (in L/day) and an average water intake rate $\hat{\omega }$ (in L/day), serves as a baseline and is typically set at 2 L/day to align with US standards. When open lower and upper intervals are available, we have:

(8) $$d_{i}=\left\{\begin{array}{c} \frac{1}{2}\cdot \delta_{0}\cdot \left(\frac{\omega }{\hat{\omega }}\right)\;\;\;\mathrm{for}\;i=0\\ \frac{1}{2}\cdot \left(\delta_{i}^{*}+\delta_{i}^{◇}\right)\cdot \left(\frac{\omega }{\hat{\omega }}\right)\;\mathrm{for}\;i\neq 0\wedge i\neq G-1\\ \frac{3}{2}\cdot \delta_{G-1}\cdot \left(\frac{\omega }{\hat{\omega }}\right)\;\;\mathrm{for}\;i=G-1 \end{array}\right.$$

If no open‐ended interval were present, the middle formula $d_{i}=2^{-1}\;\cdot \left(\delta_{i}^{*}+\delta_{i}^{◇}\right)\cdot \left(\omega /\hat{\omega }\right)$ can be used for all dose groups. Eventually, in case of epidemiological studies reporting medians instead of intervals, the above formula can be simplified to $d_{i}=\bar{\delta_{i}}\cdot \left(\omega /\hat{\omega }\right)$, where $\bar{\delta_{i}}$ represents the median dose of a given exposure group.

#### 2.3.4 Definitions of benchmark response

To facilitate an effective comparison of two modeling methods for BMD estimation using case‐control epidemiological studies, equivalent benchmark responses (BMRs) should be adequately defined for dichotomous and continuous data to minimize the impact caused by inconsistency in BMR definition. Since background exposure always exists in epidemiological studies, the reference group (typically the lowest exposure group) is a more suitable choice as a reference for BMD calculation rather than the control group (i.e., the dose level is zero) used in toxicological studies. To determine equivalent BMRs, we first choose several BMR levels for the dichotomous data, then estimate the counterpart BMRs for the continuous data.

The BMR is well defined for dichotomous data. In this study, we applied the BMR definition based on extra risk and set the value at 0.1% (low level), 0.5% (medium level), and 1% (high level), that is, three levels for comparison purposes. An important reason for choosing the BMR in this range is that, using the effective counts of lung cancer, we estimated for the selected studies, as an example, the BMRs can lead to approximately 1–20 extra cases in a 1 million population at the BMD level, which is a reasonable protective goal. Given the BMR definition, the BMD can be expressed as:

(9) $$\mathrm{BM}R_{D}=\frac{f\left(\mathrm{BMD}\right)-f\left(\mathrm{ref}\right)}{1-f\left(\mathrm{ref}\right)}$$

where, *f*(ref) is the response rate at the reference exposure group and *f*(BMD) is the response rate at the BMD exposure level. *f*(∙) represents a dichotomous dose–response model. The numerator *f*(BMD)‐*f*(ref) calculates the difference in risk between the BMD exposed and background exposed groups. This equation essentially estimates the excess risk attributable to the exposure at BMD level by dividing the difference in risk (i.e., the numerator) by the complement of the risk in the background exposed group (i.e., the denominator).

On the other hand, for continuous data, we applied the central tendency‐based definition to define the BMR, that, the BMD is the exposure level where the corresponding central tendency of the response (i.e., OR in this case) has changed by a certain amount (i.e., BMR*_C_*). The BMD calculation function can be expressed as:

(10) $$\;\mathrm{BM}R_{C}=\frac{g\left(\mathrm{BMD}\right)-g\left(\mathrm{ref}\right)}{g\left(\mathrm{ref}\right)}\;\;$$

where *g*(ref) is the OR at estimated OR at the reference exposure level and *g*(BMD) is the OR at the BMD exposure level. The *g*(∙) is a continuous dose–response model. For the case of modeling OR as continuous data, *g*(ref) serves as the reference point representing the risk level in the reference exposure group, while the numerator *g*(BMD)−*g*(ref) estimates the difference between the groups exposed at the BMD and background level, respectively. Equation (10) expresses the change in OR to the background level, providing a quantity with which to measure the relative change in risk associated with the BMD exposure.

To calculate an equivalent BMR*_C_*, we made two assumptions: (1) the OR and relative risk were generally similar for low‐prevalence diseases (e.g., bladder cancer used as an example in this study); and (2) the incidence rate at the BMD level in the scenario of OR as continuous data was equal to the incidence rate at the BMD level in the case of dichotomous data, therefore, *g* (BMD) = *f(BMD)*/*f*(ref). To avoid unwanted interruptions from model fitting process, the conversion from BMR*_D_* to the equivalent BMR*_C_* needs to be completed before the fitting and BMD estimation. Consequently, we directly used input dose–response data to calculate the quantities, for example, *g*(ref) is 1 (because OR is 1 at the reference group) and *f*(ref) is the incidence rate at the reference exposure group estimated by dividing the effective counts of cases by the total number of subjects. By substituting Equations (9) and (10) to an equation ensuring the equivalence of BMR definitions, we get Equation (11) as shown below:

(11) $$\mathrm{BM}R_{C}+1=\frac{f\left(\mathrm{BMD}\right)}{f\left(\mathrm{ref}\right)}=\frac{\mathrm{BM}R_{D}\times \left(1-f\left(\mathrm{ref}\right)\right)+f\left(\mathrm{ref}\right)}{f\left(\mathrm{ref}\right)}$$

Using Pu et al. (2007) as an example, there are 18 and 110 effective counts of cases and non‐cases, respectively indicating *f*(ref) = 18/128, so BMR*_C_* is about 0.6% when BMR*_D_* = 0.1%. Such conversion should be performed for each epidemiological dataset considered in this comparison study.

## 3 RESULTS

The results of the study are presented in three parts in this section, including (1) the input data for BMD modeling obtained after the data pre‐treatment; (2) BMD estimation using the Bayesian BMD analysis approach; and (3) the comparison of the BMD estimates using the two strategies discussed in Section 2.

For all the case‐control studies considered in this study, they were separated and summarized in Tables 2 and 3 according to the endpoints (i.e., bladder cancer and lung cancer). The estimated effective number of cases and of subjects together with the adjusted exposure midpoints are listed in the tables. These computed values were derived from the raw data, such as the exposure concentration, adjusted OR, and raw cases and non‐cases, reported in the tables as well. The water intake rate shown in the tables was used to calculate the adjusted exposure midpoints as described in Tsuji et al. (2019) and Lynch et al. (2017). Furthermore, as detailed in the caption of Table 2, for the data originated from Taiwan, we employed an up‐to‐date water intake rate value (see US Environmental Protection Agency (US EPA, 2023)), different from what was originally used by De Pretis et al. (2024).

**FIGURE 3:**
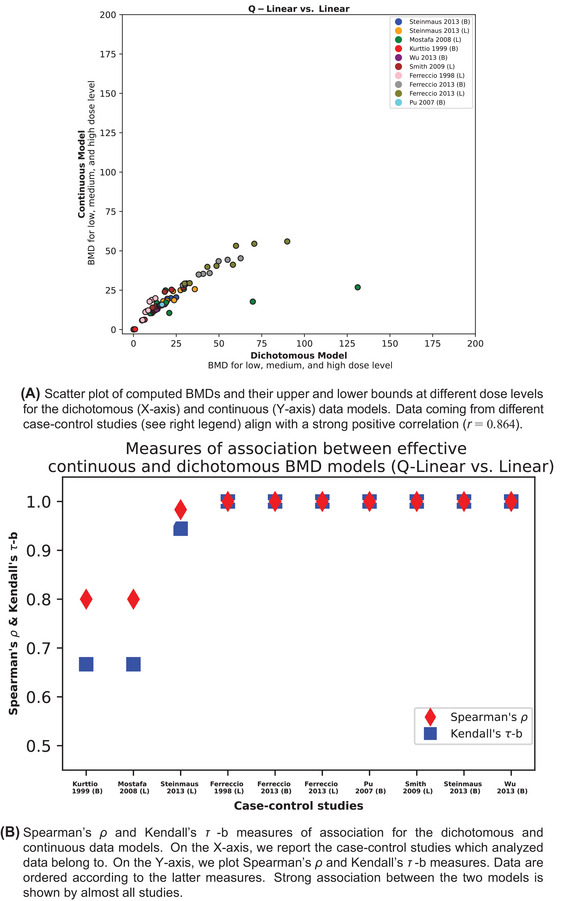
Comparison of dichotomous and continuous data models via Quantal‐Linear and Linear dose‐response models. Panel (a): BMDs and their lower and upper bounds computed via dichotomous and continuous data models. Panel (b): Measures of associations of the latter models.

**FIGURE 4:**
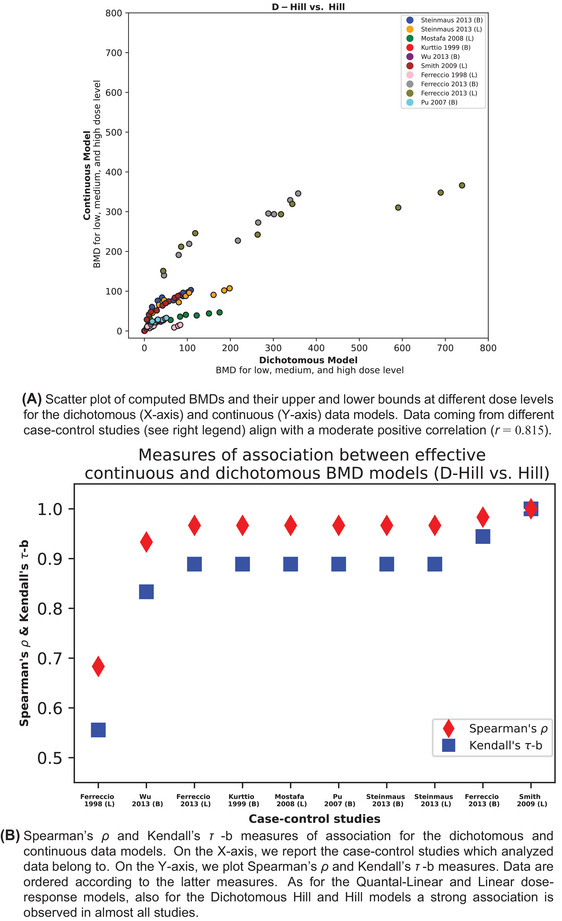
Comparison of dichotomous and continuous data models via Dichotomous Hill and Hill dose‐response models. Panel (a): BMDs and their lower and upper bounds computed via dichotomous and continuous data models. Panel (b): Measures of associations of the latter models.

With the data shown in Tables 2 and 3 as input data, we conducted a Bayesian BMD analysis using the models for dichotomous and continuous data as discussed in the previous section. Also as mentioned in the previous section, one simple model and one complex model were employed for BMD estimation for both data types. For dichotomous data, the Quantal‐Linear and Dichotomous‐Hill models are the simple and complex model, respectively, while the counterparts for continuous data are the Linear and Hill models. As described in Subsection 2.3.4, we set the BMR at 0.1%, 0.5%, and 1% for all datasets when the modeling approach for dichotomous data was applied, and the corresponding BMRs for continuous data were calculated. The complete results of the Bayesian BMD analysis together with the BMR information are reported in the Supplementary Material. We present the comparison between these two types of BMD modeling approaches, that is, the BMDs and their lower and upper bounds for a restriction to Linear vs. Quantal‐Linear model and Hill vs. Dichotomous‐Hill model, in the scatterplots reported for each case‐control study in Figures 3a and 4a.

Related to the third point concerning the performance measurement of both models, we first calculate the correlation coefficient to compare the corresponding BMD estimates (including the median, lower, and upper bound) obtained from these two modeling strategies considered using the same dataset. These BMD estimates obtained from the simple model and complex model are visualized in Figures 3a and 4a with *r *= 0.895 and *r *= 0.857, respectively. We then employed two common measures of rank correlation to quantify the statistical non‐independence between the rankings of two variables over the same dose–response models, namely the Kendall's *τ* ‐b and Spearman's *ρ* coefficients. Both measures range from −1 to 1. Positive values indicate how well the relationship between two variables can be described by an increasing monotonic function (Spearman's *ρ*) and how effectively a direct ordinal association between two measured quantities can be established (Kendall's *τ* ‐b). The results of such an analysis were visualized in Figures 3b and 4b. These figures show comparable results with an average Spearman's rank correlation coefficient accounting to 0.936 ± 0.043 (computed via Fisher's Z transformation) and an average Kendall rank correlation coefficient equal to 0.864 ± 0.045.

## 4 DISCUSSION

In this paper, following a previous work by De Pretis et al. (2024) focusing on prospective cohort study data, we aimed to identify a generalizable and standardizable BMD modeling method for case‐control epidemiological study data. Two modeling strategies were proposed and compared: that is, the modeling of the epidemiological data extracted from case‐control studies as dichotomous and continuous data, respectively. When modeling the case‐control study data as dichotomous dose–response data, we converted commonly reported information (e.g., adjusted odds ratio) in published studies to “effective counts” of cases and non‐cases, which essentially represent the incidence only caused by exposure to the study chemical (without impact from confounders). This approach appropriately weighs each exposure group while accounting for the influence of confounders on the incidence rate. Conversely, when modeling commonly reported adjusted odds ratio as continuous dose–response data, a minor data preprocessing needs to be completed to transform the reported ORs (typically median and 95^th^ confidence interval) to the format of mean and standard deviation. This method shows how health effects can be impacted by the exposure compared to the reference group.

It is important to emphasize that the modeling methods presented in this study do not involve direct analysis of raw epidemiological data. Instead, we rely on published data that has undergone appropriate processing and adjustment. While we used epidemiological cancer endpoints for demonstration purposes, this BMD modeling approach is applicable to a wide range of health effects other than cancer. For lung cancer and bladder cancer induced by exposure to iAs, numerous studies (Tsuji et al, 2019, Shao et al, 2021) have demonstrated the existence of an exposure “threshold” from both biological and statistical perspectives. However, it is not our intention to suggest that the estimated BMDL values should be used to develop a reference dose (RfD) or cancer slope factor (CSF) for iAs. Instead, we want to mention that the extrapolation of the derived epidemiological BMD to lower doses, or the methods for conducting low‐dose extrapolation for epidemiological BMD, is important but falls outside the scope of this study.

The conversion of published OR dose–response data ensures they align with the standardized format required for BMD analysis using continuous data. This allows for seamless applications of commonly used dose–response models and standard BMR definitions and settings in epidemiological BMD analysis. To emphasize and guarantee an effective comparison between different data structures for BMD estimation (i.e., modeling case‐control dose–response data as dichotomous vs. continuous data), we implemented strategies to minimize the influence of other factors on the BMD estimates: (1) calculating equivalent BMR values for continuous data based on the specified BMR values for dichotomous data, using incidence rates as a reference; and (2) selecting comparable dose–response models for the two data types, such as Quantal‐Linear vs. Linear and Dichotomous‐Hill vs. Hill models.

The estimated BMD, BMDL, and BMDU values from these two modeling strategies were analyzed for correlation. The correlation coefficients were 0.895 and 0.857 when the simple and complex dose–response models were employed, respectively, indicating that the BMD estimates from these two modeling approaches are relatively consistent. The slightly lower correlation coefficient in the complex model situation may be due to larger estimation uncertainty in model fitting, similar to the findings by De Pretis et al. (2024) when dealing with prospective cohort study data. The compatible BMD estimates from the two modeling strategies are further confirmed by the values of Kendall's *τ ‐b* and Spearman's *ρ* coefficients.

From a theoretical standpoint, modeling case‐control study data as either dichotomous or continuous response for BMD estimation are generally consistent. However, in practice, modeling OR as a continuous response is more advantageous. This is because converting confidence intervals into mean and standard deviation is much more straightforward than employing numerical methods to derive effective counts. As a result, modeling OR as continuous data simplifies the implementation process in BMD modeling tools. Notably, these findings align with those observed for RRs.This latter result also aligns with the previous work by De Pretis et al. (2024), which integrated epidemiological data from prospective cohort studies into toxicological risk assessment. This consistency with prior studies underscores the robustness and generalizability of our proposed BMD modeling methods. It confirms that our methods side with established practices in BMD analysis, further validating our approach.

## CONFLICT OF INTEREST STATEMENT

The authors declare no conflict of interest.

## SUPPORTING INFORMATION

Additional supporting information can be found online in the supplementary material downloadable at the end of this article.

## Supporting information



SUPPORTING INFORMATION

## Data Availability

Data sharing not applicable to this article as no new datasets were generated or analysed during the current study.
